# Cannabinoid CB_1_ Receptors in the Intestinal Epithelium Are Required for Acute Western-Diet Preferences in Mice

**DOI:** 10.3390/nu12092874

**Published:** 2020-09-20

**Authors:** Bryant Avalos, Donovan A. Argueta, Pedro A. Perez, Mark Wiley, Courtney Wood, Nicholas V. DiPatrizio

**Affiliations:** 1 Division of Biomedical Sciences, School of Medicine, University of California, Riverside, Riverside, CA 92521, USA; baval002@ucr.edu (B.A.); daarguet@hs.uci.edu (D.A.A.); Pedro.Perez@medsch.ucr.edu (P.A.P.); Mark.Wiley@medsch.ucr.edu (M.W.); cwood019@ucr.edu (C.W.); 2 Department of Medicine, School of Medicine, University of California, Irvine, Irvine, CA 92697, USA

**Keywords:** endocannabinoid, cannabinoid CB_1_ receptor, gut–brain, intestine, western diet, preference

## Abstract

The endocannabinoid system plays an important role in the intake of palatable food. For example, endocannabinoid signaling in the upper small-intestinal epithelium is increased (i) in rats after tasting dietary fats, which promotes intake of fats, and (ii) in a mouse model of diet-induced obesity, which promotes overeating via impaired nutrient-induced gut–brain satiation signaling. We now utilized a combination of genetic, pharmacological, and behavioral approaches to identify roles for cannabinoid CB_1_Rs in upper small-intestinal epithelium in preferences for a western-style diet (WD, high-fat/sucrose) versus a standard rodent diet (SD, low-fat/no sucrose). Mice were maintained on SD in automated feeding chambers. During testing, mice were given simultaneous access to SD and WD, and intakes were recorded. Mice displayed large preferences for the WD, which were inhibited by systemic pretreatment with the cannabinoid CB_1_R antagonist/inverse agonist, AM251, for up to 3 h. We next used our novel intestinal epithelium-specific conditional cannabinoid CB_1_R-deficient mice (IntCB_1_−/−) to investigate if intestinal CB_1_Rs are necessary for WD preferences. Similar to AM251 treatment, preferences for WD were largely absent in IntCB_1_−/− mice when compared to control mice for up to 6 h. Together, these data suggest that CB_1_Rs in the murine intestinal epithelium are required for acute WD preferences.

## 1. Introduction

Humans and other mammals, when given a choice, generally prefer food that contains fats, sugars, or a combination of both [1]. Homeostatic and hedonic feeding are controlled by diverse, albeit overlapping, neural and molecular signaling pathways throughout the brain, including those regulated by the endocannabinoid (eCB) system [2,3,4,5]. Recent studies, however, suggest important roles for the peripheral eCB system in energy homeostasis and intake of palatable food [6,7,8,9,10,11,12,13,14,15,16,17,18,19,20,21,22,23,24,25,26,27,28,29]. For example, we reported that tasting dietary lipids was sufficient to increase levels of eCBs in the rat upper small-intestinal epithelium, which required an intact vagus nerve, and pharmacological inhibition of cannabinoid subtype-1 receptors (CB_1_Rs) in the periphery blocked consumption of lipids [14,15]. Moreover, levels of eCBs in the upper small-intestinal epithelium were increased in mice maintained for eight weeks on a western-style diet high in fat and sugar (WD) when compared to mice fed a standard diet low in fat and sugar, and pharmacological inhibition of CB_1_Rs in the periphery blocked overeating associated with WD-induced obesity [17].

Nutrients are sensed by gustatory cells in the oral cavity and enteroendocrine cells in the intestinal epithelium. In response, these cells release several satiation- and satiety-related molecules that communicate with the brain via a mechanism that includes the afferent vagus nerve [30,31,32,33,34,35,36,37,38]. We recently reported that eCB signaling in the gut controls nutrient-induced release of satiation peptides [16]. Gene transcripts for CB_1_Rs were enriched in a subpopulation of enteroendocrine cells in the upper small-intestinal epithelium that secrete the satiation peptide, cholecystokinin [16,39]. Notably, the ability for nutrients to stimulate an increase in levels of circulating cholecystokinin was impaired in mice fed WD for eight weeks when compared to lean control mice, and pharmacological inhibition of overactive eCB signaling at peripheral CB_1_Rs in mice fed WD restored the ability for nutrients to induce release of cholecystokinin [16]. Furthermore, the appetite-suppressing effects of peripheral CB_1_R inhibition in mice maintained on WD were attenuated by co-treatment with an antagonist for cholecystokinin-A receptors [16], which are expressed by sensory vagal neurons and other organs [40]. Collectively, these studies suggest that eCB signaling in upper small-intestinal epithelium is dysregulated in WD-induced obese mice and promotes overeating by a mechanism that includes blocking nutrient-induced gut–brain satiation signaling.

In the current study, we used a novel conditional intestinal epithelium-specific CB_1_R-deficient mouse model to investigate if CB_1_Rs in the intestinal epithelium are required for WD preferences.

## 2. Materials and Methods

### 2.1. Animals

C57BL/6Tac male mice (Taconic, Oxnard, CA, USA) or transgenic mice (described below in Transgenic Mouse Generation) 8–10 weeks of age were group-housed with ad-libitum access to a standard rodent laboratory diet (SD; Teklad 2020x, Envigo, Huntingdon, UK; 16% kcal from fat, 24% kcal from protein, 60% kcal from carbohydrates) and water throughout all experiments. Mice were maintained on a12-h dark/light cycle beginning at 1800 h. All procedures met the U.S. National Institute of Health guidelines for care and use of laboratory animals and were approved by the Institutional Animal Care and Use Committee (IACUC Protocol 20200023) of the University of California, Riverside.

### 2.2. Transgenic Mouse Generation

Conditional intestinal epithelium-specific CB_1_R-deficient mice (Cnr1^tm1^.^1 mrl^/vil-cre ERT2) were generated by crossing Cnr1-floxed mice (Cnr1^tm1^.^1 mrl^; Taconic, Oxnard, CA, USA; Model # 7599) with Vil-CRE ERT2 mice donated by Dr. Randy Seeley (University of Michigan, Ann Arbor, MI, USA) with permission from Dr. Sylvie Robin (Curie Institute, Paris, France). Cre recombinase expression in the intestinal epithelium is driven by the villin promotor, which allows for conditional tamoxifen-dependent Cre recombinase action to remove the *Cnr1* gene from these cells, as described by el Marjou et al., [41]. When compared to other mouse lines that exhibit extra-intestinal expression of CRE recombinase, the Vil-CRE ERT2 mice used in our studies show selective expression in the intestinal epithelium with scattered expression in the testis [42]. Cnr1^tm1^.^1 mrl^/vil-cre ERT2 mice used in these experiments are referred to as IntCB_1_−/−, and Cnr1^tm1^.^1 mrl^ control mice (lacking Cre recombinase) are referred to as IntCB_1_+/+. Tail snips were collected from pups at weaning and DNA was extracted and analyzed by conventional PCR using the following primers (5′-3′): GCAGGGATTATGTCCCTAGC (CNR1-ALT), CTGTTACCAGGAGTCTTAGC (1415-35), GGCTCAAGGAATACACTTATACC (1415-37), GAACCTGATGGACATGTTCAGG (vilcre, AA), AGTGCGTTCGAACGCTAGAGCCTGT (vilcre, SS), TTACGTCCATCGTGG-ACAGC (vilcre, MYO F), TGGGCTGGGTGTTAGCCTTA (vilcre, MYO R).

### 2.3. Western Diet Preference Test

Mice were single-housed in two-hopper feeding chambers (TSE Systems, Chesterfield, MO, USA) for five days to acclimate, and received ad-libitum access to SD and water throughout behavioral testing. At the time of testing, mice were given access for the first time to the hopper containing Western Diet (WD; Research Diets D12079B, New Brunswick, NJ, USA; 40% kcal from fat, 17% kcal from protein, 43% kcal from carbohydrates as mostly sucrose). Food weights were measured in real time and recorded every minute using Phenomaster software (TSE Systems). Preferences for WD versus SD (% total kcals from WD), total caloric intake of each diet (kcals), average meal size of each diet (kcals), and meal frequency were calculated from recorded data, beginning one hour before the dark cycle (1700 h). The criteria for a meal was consumption of a minimum of 0.1 g of food with an inter-meal interval less than 30 min.

### 2.4. Chemical Preparation and Administration

IntCB_1_−/− and IntCB_1_+/+ mice were administered tamoxifen (Intraperitoneal, 40 mg per kg) every 24 h for five consecutive days. Tamoxifen (Sigma-Aldrich, St. Louis, MO, USA) was dissolved in corn oil at a concentration of 10 mg per mL then stored at 37 °C protected from light until administration. Tamoxifen in corn oil was placed in a bath sonicator for 10 min prior to administration. Mice were group housed in disposable cages throughout the injection window and for a 3-day post-injection period. The CB_1_R antagonist/inverse agonist, AM251 (Tocris, Minneapolis, MN, USA), was administered (Intraperitoneal, 3 mg per kg per 2 mL) 30 min prior to testing. The vehicle consisted of 7.5% dimethyl sulfoxide (DMSO, Sigma-Aldrich, St. Louis, MO, USA), 7.5% Tween 80 (Chem Implex Intl Inc., Wood Dale, IL, USA), and 85% sterile saline.

### 2.5. Immunohistochemistry

Proximal small intestinal tissue was collected from IntCB_1_−/− and IntCB_1_+/+ control mice 7 days after the completion of tamoxifen schedule. Tissue was flushed with ice-cold 4% paraformaldehyde/phosphate-buffered saline then fixed for 4 h at 4 °C. Cross sections of the upper small intestine were cut and frozen in embedding medium (Fisher Healthcare, Chino, CA, USA) on dry ice. Approximately 16 μm sections were obtained using a cryostat (Leica, Wetzlar, Germany) then mounted onto charged glass slides. Sections were permeabilized with 0.5% Tween20/PBS and then blocked with 0.1% Tween20 in casein solution (Thermo Fisher, Waltham, MA, USA). Primary antibodies for CB_1_Rs (kindly provided by Dr. Ken Mackie, Indiana University, Bloomington, IL, USA) raised in rabbit were diluted 1:500 in blocking buffer, slides were incubated for 1 h at room temperature. Sections were washed three times with 0.1% Tween20/PBS solution then incubated for 1 h at room temperature with goat anti-rabbit secondary antibodies conjugated with alexafluor 647. Following repeated washes, coverslips were mounted with Prolong Gold Antifade reagent with DAPI (Thermo Fisher) for nuclear counterstaining. Images were obtained at room temperature using an Axio Observer Z1 Inverted Microscope (Zeiss, Oberkochen, Germany) as previously described [16].

### 2.6. Gene Expression

Total RNA from intestinal epithelium tissue was extracted using a RNeasy kit (Qiagen, Valencia, CA, USA) and first-strand cDNA was generated using M-MLV reverse transcriptase (Invitrogen, Carlsbad, CA, USA). Areas used for tissue collection and processing were sanitized with 70% ethanol solution then treated with an RNAse inhibitor (RNAse out, G-Biosciences, St. Louis, MO, USA). Reverse transcription of total RNA was performed as previously described [16]. Quantitative RT-PCR was performed using PrimePCR Assays (Biorad, Irvine, CA, USA) with primers for CB_1_R (Cnr1), CB_2_R (Cnr2), g-protein coupled receptor 55 (Gpr55), diacylglycerol lipase alpha (Dagla), diacylglycerol lipase beta (Daglb), monoacylglycerol lipase (Mgll), alpha beta hydrolase domain containing 6 (Abhd6), N-acyl-phosphatidylethanolamine-hydrolyzing phospholipase D (Napepld), and fatty acid amide hydrolase (Faah) gene transcripts under preconfigured SYBR Green assays (Biorad, Irvine, CA, USA). Relative quantification using the delta-delta (2^−ΔΔCq^) method was used to compare changes in gene expression between IntCB_1_−/− mice and control IntCB_1_+/+ mice. Tissue specific housekeeping genes served as internal controls and were validated by verifying that expression was not affected between experimental conditions. Hprt was used as a housekeeping gene for stomach, duodenum intestinal epithelium, jejunum intestinal epithelium, ileum intestinal epithelium, small-intestinal submucosa/muscle/serosal layer, large intestinal epithelium, and liver; β-actin (Actb) as housekeeping gene for pancreas; and β2-microglobulin (B2m) as housekeeping gene for epididymal fat. Reactions were run in triplicate and values are expressed as relative mRNA expression.

### 2.7. Statistical Analysis

Data were analyzed by GraphPad Prism 8 software using unpaired Student’s *t*-tests (two-tailed) or two-way ANOVA with Holm-Sidak’s multiple comparisons post-hoc test when appropriate. Results are expressed as means ± S.E.M. and significance was determined at *p* < 0.05.

## 3. Results

### 3.1. Systemic Pharmacological Blockade of CB_1_Rs Reduces Acute Preferences for Western Diet in Mice

**Figure 1:**
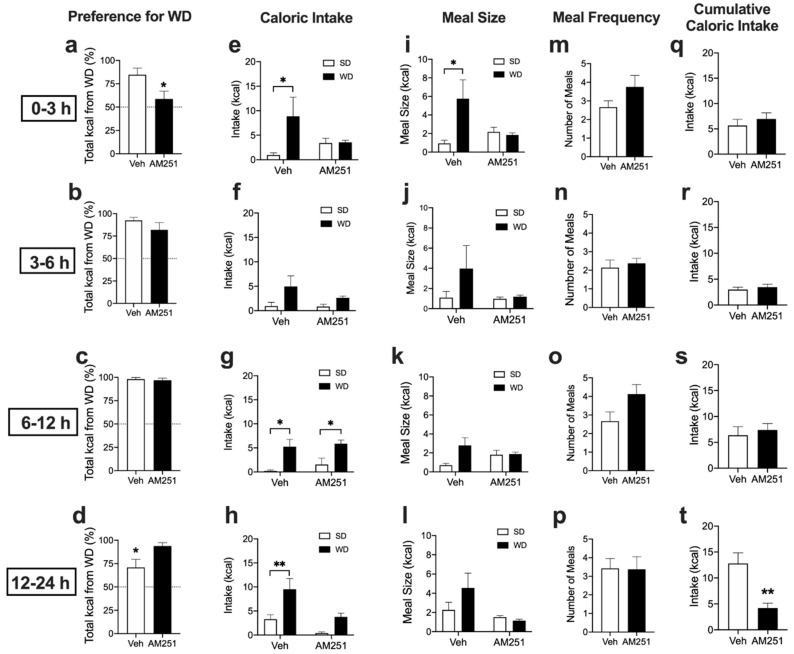
Cannabinoid CB_1_Rs control acute preferences for Western Diet. Veh = vehicle treatment; AM251 = 3mg per kg; SD = standard rodent chow; WD = western diet. Unpaired Student’s *t*-test, two-tailed (**a**–**d**,**m**–**t**); Two-way ANOVA with Holm-Sidak’s multiple comparison tests (**e**–**l**); * *p* < 0.05, ** *p* < 0.01. Results are expressed as means ± S.E.M; *n* = 7–8 per condition.

We investigated roles for cannabinoid CB_1_ receptors in preferences for Western Diet (WD). Naïve mice maintained on ad-libitum standard laboratory chow diet (SD) were administered the vehicle or the cannabinoid CB_1_R antagonist/inverse agonist, AM251 (3mg per kg), and subjected to a 24 h preference test for WD versus SD. Vehicle-treated mice displayed robust preferences for WD when compared to SD, an effect inhibited by AM251 by 3 h (Figure 1a, from 84.7 ± 7.1% total kcals from WD in vehicle-treated mice compared to 58.7 ± 8.3% in AM251-treated mice; *p* = 0.042). These effects were absent during the 3–6 h interval (Figure 1b, from 92.4 ± 3.5% total kcals from WD in vehicle-treated mice compared to 81.9 ± 8.0% in AM251-treated mice; *p* = 0.25), and the 6–12 h interval (Figure 1c from 98.0 ± 1.9% total kcals from WD in vehicle-treated mice compared to 96.9 ± 2.1% in AM251-treated mice; *p* = 0.69) after initiation of the preference test. Vehicle-treated mice displayed significant reductions in preference for WD during the 12–24 h interval when compared to AM251-treated mice, (Figure 1d, from 70.9 ± 8.7% total kcals from WD in vehicle-treated mice compared to 93.85 ± 3.57% in AM251-treated mice; *p* = 0.024). This was due to increases in SD intake in vehicle-treated mice during the 12–24 h interval (see Figure 1h) rather than an actual increase in preference for WD in AM251-treated mice.

Consistent with these data, vehicle-treated mice ate significantly more kcals from WD than from SD by 3 h (Figure 1e, *p* = 0.046), during the 6–12 h interval (Figure 1g, *p* = 0.019), and the 12–24 h interval (Figure 1h, *p* = 0.009), but not during the 3–6 h interval (Figure 1f, *p* = 0.09) after initiation of the preference test. These effects were absent in mice treated with AM251 by 3 h (Figure 1e, *p* = 0.951), during the 3–6 h interval (Figure 1f, *p* = 0.588), and the 12–24 h interval (Figure 1h, *p* = 0.151); however, mice consumed significantly more WD than SD during the 6–12 h interval (Figure 1g, *p* = 0.028). Moreover, vehicle-treated mice displayed larger meal sizes of WD versus SD by 3 h (Figure 1i, *p* = 0.028), an effect that failed to reach significance during the 3–6 h interval (Figure 1j, *p* = 0.359), the 6–12 h interval (Figure 1k, *p* = 0.42), and the 12–24 h interval after initiation of the preference test (Figure 1l, *p* = 0.396). Increases in meal size for WD by 3 h were absent in mice treated with AM251 (Figure 1i, *p* = 0.816). AM251 had no effect on meal frequency by 3h (Figure 1m, *p* = 0.189), during the 3–6 h interval (Figure 1n, *p* = 0.629), the 6–12 h interval (Figure 1o, *p* = 0.071) and the 12–24 h interval (Figure 1p, *p* = 0.95) after initiation of the preference test. In addition, there were no significant differences in total cumulative caloric intake (i.e., total kcals from WD + SD) between treatment groups by 3 h (Figure 1q, *p* = 0.501), during the 3–6 h interval (Figure 1r, *p* = 0.569), and the 6–12 h interval after initiation of the preference test (Figure 1s, *p* = 0.619). Despite large preferences for WD in AM251-treated mice during the 12–24 h interval (see Figure 1d), these mice consumed significantly less total calories during the 12–24 h interval after initiation of the preference test (Figure 1t, *p* = 0.002). Collectively, these results suggest that cannabinoid CB_1_Rs control acute preferences for WD in mice.

### 3.2. Acute Preferences for Western Diet are Absent in Mice with CB_1_R Deletion in the Intestinal Epithelium

**Figure 2:**
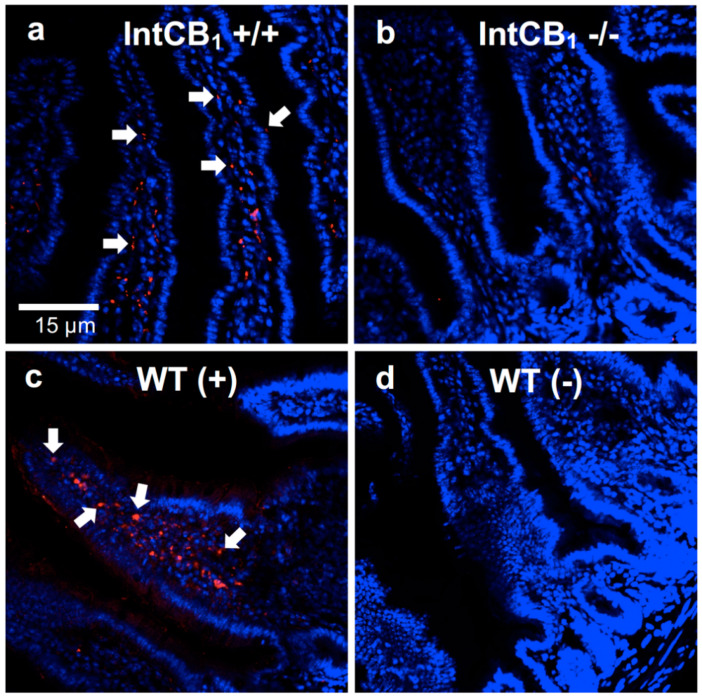
CB_1_R immunoreactivity is absent in the upper small-intestinal epithelium of conditional intestinal epithelium-specific CB_1_R-deficient mice. When compared to control mice (**a**, IntCB +/+), conditional intestinal epithelium-specific CB_1_R-null mice (**b**, IntCB_1_−/−) are deficient in immunoreactivity for CB_1_Rs in the upper small-intestinal epithelium. Wild-type C57BL/6Tac mice display immunoreactivity for CB_1_Rs in the upper small-intestinal epithelium (**c**, WT (+)), which is absent when the primary CB_1_R antibody is not included (**d**, WT(-)). White arrows point to representative red immunoreactivity for CB_1_Rs. Red = CB_1_R immunoreactivity; blue = DAPI. WT = wild-type mice. (+) = with CB_1_R primary antibody; (-) = without CB_1_R primary antibody.

Endocannabinoid signaling in the rodent upper small-intestinal epithelium is important for consumption of dietary fats based on their taste properties [14,15], re-feeding after a fast [10], and hyperphagia in a mouse model of WD-induced obesity via a mechanism that includes blocking nutrient-induced gut–brain satiation signaling [16,17]. We used our novel intestinal epithelium-specific conditional CB_1_R-deficient mice (IntCB1−/−) to probe the necessity for CB_1_Rs in the intestinal epithelium in preferences for WD. Moreover, AM251 is reported to have some off-target effects [43,44]; therefore, this mouse model allows for direct evaluation of roles for CB_1_Rs in the intestinal epithelium in these processes. CB_1_R deficiency in the intestinal epithelium of IntCB_1_−/− mice was verified by immunohistochemistry (Figure 2a–d).

**Figure 3:**
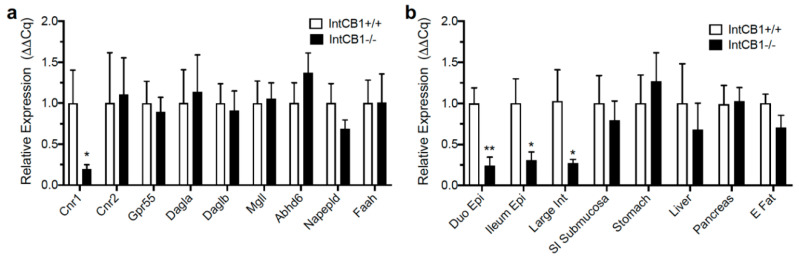
Expression of endocannabinoid system genes in conditional intestinal epithelium-specific CB_1_R-deficient mice and controls. Expression of cannabinoid CB_1_Rs (*Cnr1*) was reduced in the jejunum epithelium of conditional intestinal epithelium-specific CB_1_R deficient mice (IntCB_1_−/−) when compared to control mice (IntCB_1_+/+) (**a**), and expression of mRNA for other components of the endocannabinoid system was unaffected, including cannabinoid CB_2_Rs (*Cnr2*), g-protein coupled receptor 55 (*Gpr5*), diacylglycerol lipase alpha (*Dagla*), diacylglycerol lipase beta (*Daglb*), monoacylglycerol lipase (*Mgll*), alpha beta hydrolase domain containing 6 (*Abhd6*), N-acyl-phosphatidylethanolamine-hydrolyzing phospholipase D (*Napepld*), and fatty acid amide hydrolase (*Faah*) (**a**). IntCB_1_−/− mice, when compared to IntCB1+/+ controls, were deficient in expression of mRNA for CB_1_Rs (*Cnr1*) in the duodenum epithelium (Duo Epi), ileum epithelium (Ileum Epi), large intestine (Large Int), but not in the small-intestinal submucosa/muscle/serosal layers (SI Submucosa), stomach, liver, pancreas, and epididymal fat (E Fat) (**b**). Unpaired Student’s *t*-tests, two-tailed; * *p* < 0.05, ** *p* < 0.01. Results are expressed as means ± S.E.M; *n* = 5–8 per condition.

CB_1_R deficiency in the intestinal epithelium of IntCB_1_−/− mice was further confirmed by qRT-PCR (Figure 3a,b). IntCB_1_−/− mice, when compared to IntCB_1_+/+ controls, were deficient in expression of mRNA for CB_1_Rs (*Cnr1*) in the jejunum epithelium (Figure 3a, *p* = 0.031). Expression of mRNA for other components of the endocannabinoid system in the jejunum epithelium was unaffected, including cannabinoid CB_2_Rs (*Cnr2*; Figure 3a, *p* = 0.892), g-protein coupled receptor 55 (*Gpr55*; Figure 3a, *p* = 0.736), diacylglycerol lipase alpha (*Dagla*; Figure 3a, *p* = 0.825), diacylglycerol lipase beta (*Daglb*; Figure 3a, *p* = 0.798), monoacylglycerol lipase (*Mgll*; Figure 3a, *p* = 0.872), alpha beta hydrolase domain containing 6 (*Abhd6*; Figure 3a, *p* = 0.314), N-acyl-phosphatidylethanolamine-hydrolyzing phospholipase D (*Napepld*; Figure 3a, *p* = 0.217), and fatty acid amide hydrolase (*Faah*; Figure 3a, *p* = 0.986). In addition to the jejunum epithelium, IntCB_1_−/− mice were deficient in expression of mRNA for CB_1_Rs (*Cnr1*) in the duodenum epithelium (Figure 3b, *p* = 0.009), ileum epithelium (Figure 3b, *p* = 0.038), large intestine epithelium (Figure 3b, *p* = 0.039), but not in the small-intestinal submucosa/muscle/serosal layers (Figure 3b, *p* = 0.633), stomach (Figure 3b, *p* = 0.602), liver (Figure 3b, *p* = 0.593), pancreas (Figure 3b, *p* = 0.9), and epididymal fat (Figure 3b, *p* = 0.14).

**Figure 4:**
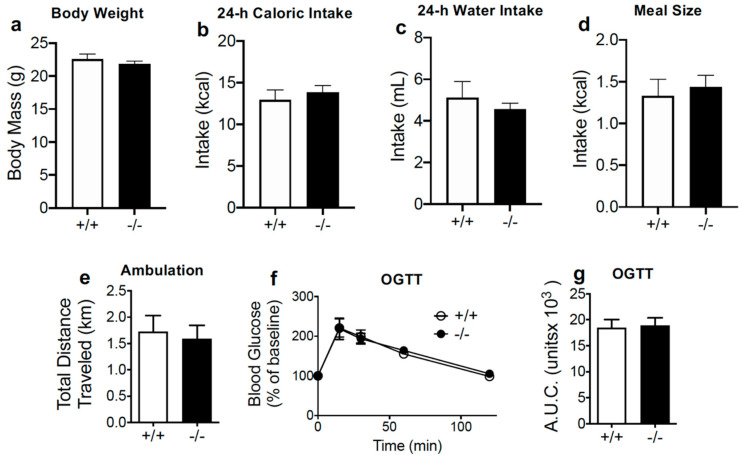
Conditional intestinal epithelium CB_1_R-deficient mice display no changes in baseline feeding parameters, motor activity, or glucose clearance. Unpaired Student’s *t*-test, two-tailed (**a**–**e**,**g**; *p* > 0.05); two-way Repeated Measures ANOVA with Holm-Sidak’s multiple comparison tests (**f**; not significant). Results are expressed as means ± S.E.M; *n* = 7–8 per condition (**a**–**e**), *n* = 3–4 (**f**,**g**). +/+ = IntCB_1_+/+ mice, −/− = IntCB_1_−/− mice; OGTT = oral glucose tolerance test; AUC = area under the curve.

IntCB_1_−/− and IntCB_1_+/+ control mice displayed similar body weights (Figure 4a, *p* = 0.404), and baseline 24-h caloric intake (Figure 4b, *p* = 0.52), 24-h water intake (Figure 4c, *p* = 0.487), average meal size (Figure 4d, *p* = 0.653), ambulation (Figure 4e, *p* = 0.741), and glucose clearance during an oral glucose tolerance test (Figure 4f,g; 15, 30, 60, 120 min, ns; total area under curve, *p* = 0.847).

**Figure 5:**
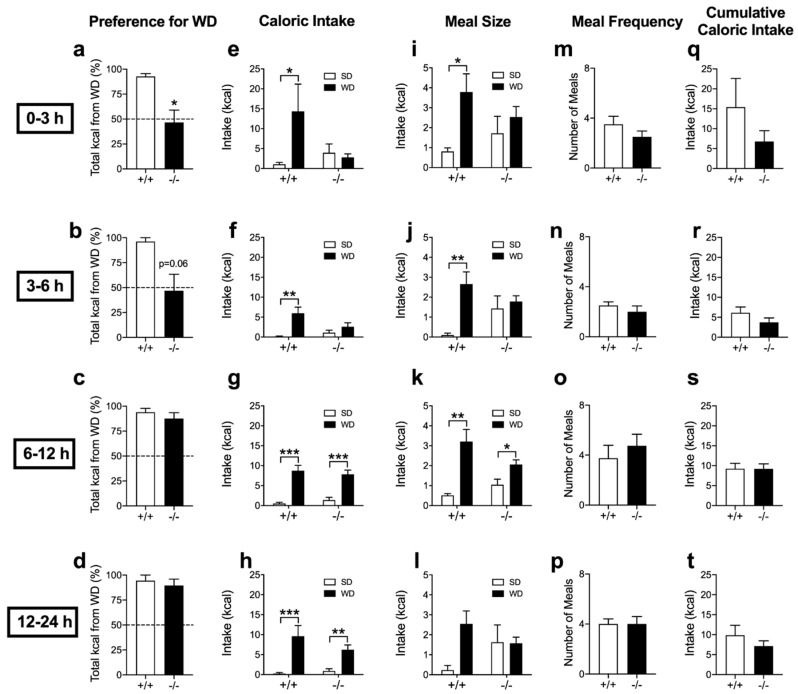
Acute preferences for western diet are absent in conditional intestinal epithelium-specific CB_1_R-deficient mice. +/+ = IntCB_1_+/+ control mice; −/− = IntCB_1_−/− mice; SD = standard rodent chow; WD = western diet. Unpaired Student’s *t*-test, two-tailed (**a**–**d**,**m**–**t**); Two-way ANOVA with Holm-Sidak’s multiple comparison tests (**e**–**l**); * *p* < 0.05, ** *p* < 0.01, *** *p* < 0.001. Results are expressed as means ± S.E.M; *n* = 4–8 per condition.

Control IntCB_1_+/+ mice displayed robust preferences for WD when compared to SD, an effect largely absent in IntCB_1_−/− mice by 3 h (Figure 5a, from 92.8 ± 2.8% total kcals from WD in IntCB_1_+/+ mice compared to 46.5 ± 12.5% in IntCB_1_−/− mice; *p* = 0.029), and approaching significance during the 3–6 h interval (Figure 5b, from 96.2 ± 3.8% total kcals from WD in IntCB_1_+/+ mice compared to 46.9 ± 16.5% in IntCB_1_−/− mice; *p* = 0.06). Preferences for WD in IntCB_1_−/− mice were not different from controls by the 6–12 h interval (Figure 5c, from 94.0 ± 3.8% total kcals from WD in IntCB_1_+/+ mice compared to 87.6 ± 5.8% in IntCB_1_−/− mice; *p* = 0.49) and the 12–24 h interval (Figure 5d, from 94.5 ± 5.5% total kcals from WD in IntCB_1_+/+ mice compared to 89.5 ± 6.4% in IntCB_1_−/− mice; *p* = 0.633) after initiation of the preference test.

Congruent with these data, control IntCB_1_+/+ mice ate significantly more kcals from WD when compared to SD by 3 h (Figure 5e, *p* = 0.02), during the 3–6 h interval (Figure 5f, *p* = 0.004), the 6–12 h interval (Figure 5g, *p* < 0.001), and the 12–24 h interval (Figure 5h, *p* < 0.001) after initiation of the preference test. These effects were absent in IntCB_1_−/− mice by 3 h (Figure 5e, *p* = 0.732), and during the 3–6 h interval (Figure 5f, *p* = 0.205); however, intakes for WD rebounded in IntCB_1_−/− mice by the 6–12 h interval (Figure 5g, *p* <0.001) and during the 12–24 h interval (Figure 5h, *p* = 0.002). Moreover, IntCB_1_+/+ mice displayed larger meal sizes of WD versus SD by 3 h (Figure 5i, *p* = 0.031), during the 3–6 h interval (Figure 5j, *p* = 0.005), and the 6–12 h interval (Figure 5k, *p* = 0.002), but not during the 12–24 h interval (Figure 5l, *p* = 0.072) after initiation of the preference test. These effects were absent in IntCB_1_−/− mice by 3 h (Figure 5i, *p* = 0.404), during the 3–6 h interval (Figure 5j, *p* = 0.589), and the 12–24 h interval (Figure 5l, *p* = 0.958); however, meal size of WD versus SD was increased in InCB_1_−/− mice during the 6–12 h interval (Figure 5k, *p* = 0.044). No significant changes were found in meal frequency for IntCB_1_−/− mice when compared to IntCB_1_+/+ controls by 3 h (Figure 5m, *p* = 0.239), during the 3–6 h interval (Figure 5n, *p* = 0.49), the 6–12 h interval (Figure 5o, *p* = 0.521), and the 12–24 h interval (Figure 5p, *p* = 0.99) after initiation of the preference test. In addition, no significant changes were found in total cumulative caloric intake (i.e., total kcals from WD + SD) for IntCB_1_−/− mice when compared to IntCB_1_+/+ controls by 3 h (Figure 5q, *p* = 0.196), during the 3–6 h interval (Figure 5r, *p* = 0.233), the 6–12 h interval (Figure 5s, *p* = 0.974), and the 12–24 h interval (Figure 5t, *p* = 0.305) after initiation of the preference test.

## 4. Discussion

We report that acute preferences for WD (i) were inhibited by global pharmacological blockade of CB_1_Rs, and (ii) were largely absent in mice conditionally deficient in CB_1_Rs selectively in the intestinal epithelium. These results suggest that CB_1_Rs in the intestinal epithelium are required for acute WD preferences in mice. Moreover, these studies expand our understanding of critical pathways for gut–brain communication in the control of preferences for palatable foods.

Dietary components are detected by receptors located throughout the oral cavity [36] and intestinal epithelium [45], which provide feedback associated with the nutritional content of food and contribute to determination of food preferences. For example, we reported that tasting dietary unsaturated lipids—but not sugar or protein—triggered production of endocannabinoids in the rat upper small-intestinal epithelium, and pharmacological inhibition of endocannabinoid signaling at CB_1_Rs in the periphery blocked intake and preferences for fats in a sham-feeding model [14,15]. These studies suggest that endocannabinoid signaling in the gut contributes to the positive feedback control of fat intake based on its unique taste properties. Despite localized increases of endocannabinoids selectively in the upper small-intestinal epithelium and blockade of intake following pharmacological treatment with a peripherally-restricted neutral CB_1_R antagonist, these studies were limited in their ability to identify necessity for CB_1_Rs in the intestinal epithelium in food intake and dietary preferences. To overcome these challenges and examine whether CB_1_Rs in the small-intestinal epithelium were required for WD preferences, we generated a novel conditional intestinal epithelium-specific CB_1_R-deficient mouse. Notably, the WD used in these studies is composed of 40% kcals from fats and 43% from carbohydrates, which more closely matches the 35% fat and 47% carbohydrate composition of diets in humans [46] when compared to rodent studies that routinely use high-fat test diets containing 60% kcals from fat and relatively low levels of carbohydrates (e.g., Research Diets D12492). Robust preferences found for WD in control mice, when compared to a low-fat/no-sucrose chow, were largely absent in IntCB_1_−/− mice during the first 12 h of preference testing. In addition, these effects were mimicked by systemic treatment with the globally acting CB_1_R antagonist/inverse agonist, AM251, in wild-type mice. Collectively, these results provide evidence of a critical role for CB_1_Rs in the rodent intestinal epithelium in acute preferences for food containing high levels of fats and sugars. Humans also display robust preferences for food that contains high levels of fats and sugar [1], and consumption of palatable food was associated with elevated levels of circulating endocannabinoids in humans [21]. It remains to be determined if consumption of palatable food in humans is controlled by gut–brain endocannabinoid signaling in a similar manner to rodents.

The specific mechanism(s) underlying intestinal epithelium CB_1_R-mediated preferences for WD are unknown but may include CB_1_R control of gut–brain signaling. We reported that hyperphagia and increased meal size associated with WD-induced obesity in mice are dependent on (i) elevated levels of endocannabinoids in the upper small-intestinal epithelium and (ii) CB_1_R-mediated inhibition of nutrient-induced signaling of the satiation peptide, cholecystokinin [16]. Cholecystokinin is secreted from enteroendocrine cells in the upper small-intestinal epithelium when nutrients arrive in the lumen, and transmits satiation signals to the brain by interacting with cholecystokinin A receptors on the afferent vagus nerve [30,40,47,48,49,50] and possibly the brain [51,52]. Bohorquez and colleagues recently characterized enteroendocrine cells (i.e., neuropods) in the mouse intestinal epithelium that form functional synapses with afferent vagal fibers [33]. Neuropods sense nutrients in the lumen and in response, release glutamate and cholecystokinin, which activate afferent vagal neurons in a coordinated manner [53]. Moreover, afferent vagal neurons participate in reward-related behaviors—including flavor and place preferences—and control dopamine outflow in the mouse striatum [50]. Notably, however, studies suggest that the afferent vagus nerve is required for nutrient-induced negative feedback from the gut associated with satiation and satiety, but is dispensable for positive feedback (i.e., appetition [54]) associated with nutrient reinforcement and flavor-nutrient preference conditioning [55]. Nonetheless, it is possible that CB_1_Rs in the intestinal epithelium participate in preferences for WD by a mechanism that includes control of nutrient-induced, neuropod-mediated, afferent vagal activity and recruitment of brain reward circuits. A direct test of this hypothesis and evaluation of distinct roles for intestinal CB_1_Rs in satiation versus appetition remains for future studies.

We propose that CB_1_Rs indirectly regulate afferent vagal activity by controlling nutrient sensing and release of satiation peptides from enteroendocrine cells in the small-intestinal epithelium that directly interact with the afferent vagus nerve [16,39]. Recent studies also suggest that CB_1_Rs in the mouse stomach participate in alcohol intake by controlling formation of the appetite-stimulating hormone, ghrelin, which interacts with ghrelin receptors on afferent vagal fibers [56]. In addition to these indirect mechanisms, CB_1_Rs may also directly control afferent vagal neurotransmission and food intake [57]. For example, Burdyga and colleagues reported that fasting was associated with increased expression of CB_1_Rs in the rat vagal afferent neurons [58]. Refeeding or administration of cholecystokinin rapidly reversed fasting-induced expression of CB_1_Rs [58], which was also blunted in rats maintained on a high-fat diet [59]. In addition, administration of ghrelin blocked the effects of refeeding on CB_1_R expression [60]. Moreover, Christie and colleagues reported that low and high concentrations of methanandamide—a stable analog of anandamide—differentially modified mechanosensitivity of mouse gastric vagal afferents in vitro via a mechanism that included CB_1_Rs, TRPV1, and ghrelin receptors [61], and these effects were dysregulated in mice fed a high-fat diet for 12 weeks [62]. These studies suggest that CB_1_Rs on the afferent vagus nerve may participate in gut-brain signaling important for food intake and energy balance. Interestingly, mice with genetic deletion of CB_1_Rs on afferent vagal neurons displayed no changes in body weight or food intake, irrespective of test diet (i.e., standard versus high-fat), which suggests that vagal CB_1_Rs may not be necessary for long-term maintenance of body weight and feeding [63]. Further investigations are necessary to expand our understanding of physiological roles for the endocannabinoid system in vagal afferent neurons.

It is noteworthy that attenuation of preferences for WD were limited to the first 3 h in AM251-treated wild-type mice and the first 6 h in IntCB_1_−/− mice when compared to vehicle and IntCB_1_+/+ mice, respectively. It is plausible that restricted temporal effects of AM251 in wild-type mice reflect the pharmacokinetic properties of this compound, which displays a half-life of 22 h in rats [64]. IntCB_1_−/− mice, however, displayed a similar restriction of preferences for WD, albeit to the first 6 h of the test when compared to control mice. The mechanism(s) in this restricted response to early time points remains unknown but may reflect a circadian pattern of activity or expression of the endocannabinoid system in the intestinal epithelium that controls gut–brain signaling important for food intake. A direct examination of roles for intestinal CB_1_Rs in the circadian control of food intake remains for future studies. Moreover, post-prandial cues at later time-points may provide compensatory feedback and reinforcement and restore preferences for WD in the absence of CB_1_Rs in the intestinal epithelium. One candidate in this proposed mechanism is the satiety factor oleoylethanolamide, which is synthesized in the intestinal epithelium from dietary fats and controls food intake and possibly reward through a mechanism that requires peroxisome proliferator-activated receptor α (PPARα) and the afferent vagus [65,66,67]. Studies examining interactions between orexigenic endocannabinoid and anorexic oleoylethanolamide signaling pathways in acute and long-term dietary preferences remain for future inquiry.

In summary, these studies extend our understanding beyond central roles for the endocannabinoid system in intake and reward value of palatable food [68,69,70,71,72,73,74,75,76,77,78,79,80,81,82,83,84], and provide evidence that CB_1_Rs in the intestinal epithelium are an integral component of a gut–brain axis that controls dietary preferences. Future studies will be important to elucidate (i) specific mechanism(s) of intestinal CB_1_R-mediated preferences for palatable food, (ii) roles for CB_1_Rs in the intestinal epithelium in recruitment of brain reward circuits and the “wanting” or “liking” of palatable food [85], (iii) roles for intestinal CB_1_Rs in satiation versus appetition, (iv) interactions between CB_1_R and PPARa signaling pathways in preferences for palatable food, (v) roles CB_1_Rs in the intestinal epithelium in development and maintenance of diet-induced obesity, (vi) physiological roles for CB_1_Rs on vagal neurons, and (vii) possible circadian fluctuations in expression and function of the endocannabinoid system in the gut and its relationship with feeding behavior.
